# Multijurisdictional Approach to Biosurveillance, Kansas City

**DOI:** 10.3201/eid0910.030060

**Published:** 2003-10

**Authors:** Mark A. Hoffman, Tiffany H. Wilkinson, Aaron Bush, Wayne Myers, Ron G. Griffin

**Affiliations:** * Cerner Corporation, Kansas City, Missouri, USA; † Missouri Health Department, Kansas City, Missouri, USA

## Abstract

An electronic reporting system for a network of 22 laboratories was implemented in Kansas City, Missouri, with an independent organization acting as a data clearinghouse between the reporting laboratories and public health departments. The system ran in tandem with conventional reporting methods. Laboratory test orders and results were aggregated and mapped to a common nomenclature. Reports were delivered through a secure Internet connection to the Kansas City Health Department (KCHD); during the first 200 days of operation, 359 qualified results were delivered electronically to KCHD. Data were received more quickly than they were with conventional reporting methods: notification of chlamydia cases arrived 2 days earlier, invasive group A streptococcal disease cases arrived 2.3 days sooner, and salmonellosis cases arrived 2.7 days sooner. Data were more complete for all demographic fields, including address, age, sex, race, and date of birth. Two hundred fourteen cases reported electronically were not received by conventional means.

Biosurveillance is the automated monitoring of information sources of potential value in detecting an emerging epidemic, whether naturally occurring or the result of bioterrorism. Information sources that can be monitored for early warning include purchases of nonprescription medication (*1*) and symptoms reported during ambulatory care (*2*). Although these sources offer opportunities for early detection, they may also lead to high rates of false-positive reactions. A more definitive tool for biosurveillance is the electronic reporting of diagnostic results confirming the presence of a pathogen.

Heightened concerns about bioterrorism have led public health organizations to reevaluate methods used to report diseases. Currently, most healthcare providers notify public health organizations of reportable diseases by telephone, fax, or mail (*3*). These techniques generally delay the communication of confirmatory test results and notification of the appropriate public health organization (*4*). Underreporting is a major concern with traditional disease surveillance strategies (*5*); even cases of severe diseases sometimes go unreported (*6*). In addition, substantial variability exists in the completeness of the information sent to public health; initial reports often include only the test result and the patient name. They lack demographic details that are useful to public health officials, requiring them to perform followup calls to get the additional information (*7*). These delays and inconsistencies may impair the ability of public health officials to detect or respond to a bioterrorist event. One solution to these deficiencies is to use an electronic system to report disease to public health authorities.

Three approaches to electronic disease reporting are feasible. The first approach (Figure 1A) requires each healthcare provider to standardize clinical results (i.e., by using the Systematized Nomenclature of Medicine [SNOMED]) before sending results electronically to the appropriate authority. Researchers in Pittsburgh, working in an integrated delivery network that used a single data dictionary to minimize the difficulty of reconciling disparate coding systems, found this approach effective for electronic disease reporting (*8*). However, developing this type of system can be challenging because of the difficulty in updating multiple data dictionaries (translation tables that associate terms from multiple organizations). The second approach (Figure 1B) requires the use of result standardization software (which collects data from multiple sites and attempts to automatically associate terms to standard terms) at public health facilities. This approach places the responsibility for technology and personnel with the public health organizations. The third approach to electronic reporting (Figure 1C) involves an intermediary organization that aggregates the data and distributes standardized reports to public health organizations. Single jurisdiction systems using this approach have been developed in Hawaii and Indianapolis (*9*,*10*). We developed a multiple-jurisdiction data clearinghouse system (*11*) and assessed the benefits of the system in terms of timeliness, data completeness, and geographic depth of coverage.

**Figure 1:**
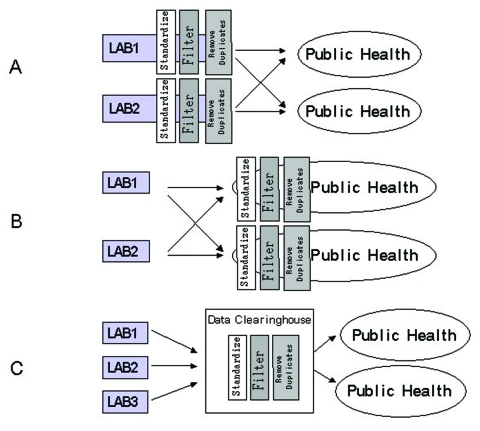
Comparison of technical approaches to biosurveillance: A) standardization, filtering, and checking for duplication done at contributor site; B) translation and checking for duplication at public health site; C) data repository.

## Methods

**Figure 2:**
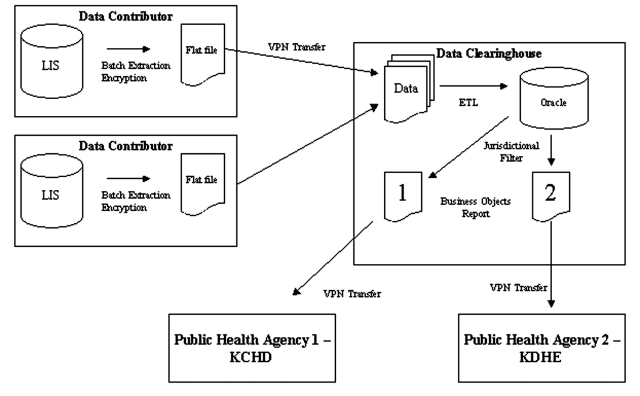
Data clearinghouse system architecture. Data are extracted from the laboratory information network at contributing sites and encrypted into a flat file. These are then delivered by virtual private network (VPN)–secured file transfer protocol to the clearinghouse where they are subjected to data warehousing processes. Jurisdictional filters are applied to the data to construct reports with data appropriate for the recipient. KCHD, Kansas City Health Department; KDHE, Kansas Department of Health and Environment.

The electronic reporting system consisted of three participating groups: data contributors, the clearinghouse, and public health organizations (Figure 2). Four nonaffiliated healthcare organizations of varying size (consisting of 2, 2, 5, and 13 facilities) participated in the system. The smallest facilities were regional care centers with 49 beds each; the largest was an urban hospital with 650 beds. All participating organizations used the same laboratory information system (LIS) (PathNet, Cerner Corp., Kansas City, MO) to document clinical microbiology results. Microbiology reports were constructed from a combination of codified entries representing the pathogen and discrete observations or free text entries added by the user. The data dictionaries and test catalogs at each organization were unique and were not referenced to standardized vocabularies. During the implementation of the electronic reporting system, the data dictionaries and test catalogs from the participating organizations were uploaded to the data clearinghouse; entries representing reportable pathogens or tests with the potential to yield reportable results were mapped to a standardized vocabulary.

We established agreements to assure security within the system. Surveillance data reported to public health organizations are exempted from the Health Insurance Portability and Accountability Act. Legal agreements designating the supplier of the clearinghouse system as a business associate with full reporting capability were established with the participating organizations. These agreements, combined with stringent access controls (physical and procedural security measures), encryption of identifiable information, and virtual private network (VPN) secured communications and ensured protection of confidentiality. Interagency agreements between public health organizations in the Kansas City area provided a further framework for the appropriate exchange of data across jurisdictional boundaries. For example, an agreement between KCHD and the state of Kansas recognized Kansas City, Missouri, as a county of Kansas for purposes of metropolitan surveillance.

**Figure 3:**
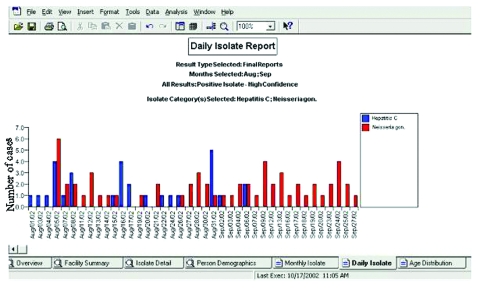
Example of a pathogen-trending report showing the trends for a user-selected set of pathogens. Other reports include facility summaries, detailed-line listings, and age trends.

Batch extraction scripts ran daily at each data contributor, pulling new laboratory test orders, laboratory results, and patient demographic information. Demographic information was encrypted before being transferred to the data clearinghouse through a VPN (Cisco Systems, San Jose, CA). The data were loaded into a data warehouse (Oracle Corp., Redwood Shores, CA) and checked for errors and duplication; data mapping was then performed (Informatica Corp., Redwood City, CA). Microbiologic results identifying reportable pathogens were mapped automatically to a common nomenclature to standardize the varying names between the participating organizations. Procedure orders (i.e., stool culture) deemed relevant to the detection of an infectious disease outbreak were also mapped to a common nomenclature. After these processing steps, the results were used to build two reports that were delivered through the Internet to KCHD using a VPN secured account. One report provided trending information on orderable procedures; the other provided results from microbiology tests (Figure 3). KCHD staff reviewed the reports using a report viewer (BusinessObjects Corp., San Jose, CA). Reports summarizing the results reported to KCHD were also delivered to the institution of origin. Jurisdictional filters were applied to deliver appropriate data to the Kansas Department of Health and Environment (these data will be analyzed separately).

We evaluated reports received through both conventional and electronic reporting between March 29, 2002, and September 2, 2002, for data completeness and timeliness. Disease reporting in Kansas City requires that public health officials know the name of the testing facility and the patient’s age, date of birth, race, sex, address, and telephone number. As each conventional or electronic report was received, KCHD documented whether each required data element was provided and documented the date on which conventional reports were received. Timeliness was determined by comparing this date with the date that a reportable pathogen was first posted to LIS.

Reports received only through electronic means were evaluated to confirm that they satisfied KCHD reporting criteria. This review included confirmation of appropriate jurisdictional concerns, origin of isolate (appropriate body site), and exclusion of false-positive results.

Geographic information system maps, showing the residential zip code of patients with reportable isolates, were delivered to KCHD by using Arc-IMS (ESRI, Redlands, CA). Users could select a pathogen and observe the zip codes of patients with confirmed cases. Geographic coverage maps were generated by using the zip codes of all patients whose results were evaluated by the system.

Critical isolate alerts were also built into the system. A single instance of *Bacillus anthracis*, *Coxiella burnetii*, *Yersinia pestis,* or any *Brucella* species would trigger alerts sent to the pagers of on-call public health officers and supervisors.

## Results

The electronic data clearinghouse study was conducted in tandem with conventional reporting at all sites. In 2002, conventional reports to KCHD originated from laboratories (52%), infection control practitioners (34%), blood centers (6%), private physicians (4%), and other sources (4%). Personnel involved in conventional reporting were generally unaware of the electronic reporting system; their management was instructed in the dual reporting requirement. Our review of reports received through both the clearinghouse and conventional reporting identified 144 isolate reports. An additional 213 cases arrived only through electronic reporting (Table 1). We reviewed the addresses of the patient and reporting laboratory to verify that a report was in the KCHD jurisdiction. Table 1 lists the specific pathogens documented through this system and the average improvement in timeliness for each pathogen. Timeliness improved for all pathogens; the improvements for chlamydia, invasive group A streptococcal infections, and salmonellosis cases were statistically significant (Table 1). One chlamydia case arrived 20 days earlier through clearinghouse reports.

**Table 1:** Comparison of reporting times between conventional and electronic reporting and evaluation of reporting coverage

Pathogen	Average days earlier^a^	Electronic and traditional^b^	Electronic only^c^	Total reports	Reporting improvement^d^
*Campylobacter* sp.	0.6	10	7	17	70%
*Chlamydia trachomatis*	2.2^e^	29	81	110	279%
*Cryptosporidium parvum*	0.0	1	-	1	-
*Escherichia coli* O157:H7	0.0	1	2	3	200%
*Giardia lamblia*	0.0	1	12	13	1,200%
*Neisseria gonorrhoeae*	0.3	50	48	98	96%
*Haemophilus influenzae* (invasive)	3.0	3	3	6	100%
Hepatitis A	0.0	1	-	1	-
Hepatitis B	0.5	4	3	7	75%
Hepatitis C	3.6	5	22	27	440%
Influenza	1.2	5	3	8	60%
Group A streptococcal infections (invasive)	2.3^f^	7	1	8	14%
*Borrelia burgdorferi*	1.3	4	3	7	75%
*Salmonella* sp.	2.7^f^	14	6	20	43%
*Shigella* sp.	0.0	2	1	3	50%
*Streptococcus pneumoniae* (invasive, drug-resistant)	8.0	1	-	1	-
*Treponema pallidum*	0.4	5	21	26	420%
*Yersinia* sp.	0.0	1	-	1	-

^a^Average days earlier was calculated by comparing the date on which the initial conventional report arrived to the date on which an electronic report was received. Only cases received by both means were used to calculate this value. ^b^Reports for these cases were received by both conventional means (mail, telephone, fax) and the laboratory information network. All reports received through traditional reporting were also received by the data clearinghouse. ^c^Reports for these cases were received only through the laboratory information network and are not included in the counts for the “electronic and traditional means” column. ^d^Received electronically only/received through both means x 100. ^e^Significant as determined by Student t test (p<0.05). ^f^Significant as determined by Wilcoxon signed rank (p<0.05).

Many case reports were only received through electronic reporting (Table 1). In particular, giardiasis and hepatitis C were underreported. Sexually transmitted diseases, including chlamydia, gonorrhea, and syphilis, were also underreported.

The data clearinghouse received all cases reported by traditional means for diseases that were consistently documented by using microbiology reports. The null value precluded the use of the Chandra Sekar-Deming capture-recapture technique used to assess reporting coverage in other electronic reporting systems (*8*,*9*). Diagnostic results verifying some diseases, especially viral diseases such as hepatitis and HIV, are sometimes documented using microbiology reports but are often documented using LIS applications from which results were not evaluated by the data clearinghouse.

Demographic information about patients was provided more often through clearinghouse reports than through reports received in tandem by traditional means. This improvement was statistically significant for patient address, race, age, date of birth, and sex, as determined by McNemar’s test (Table 2).

**Table 2:** Frequency of field completion for patient demographic and care-provider information from initial reports

	Traditional reporting (%)	Electronic reporting (%)
Facility	93	97
Address	32	79^a^
Phone	29	35
Gender	94	99^a^
Race	38	72^a^
Age	92	98
Date of birth	38	95^a^

^a^Significant as determined by McNemar’s test (p<0.05).

**Figure 4:**
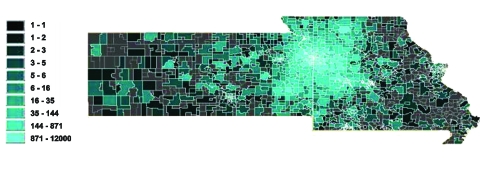
Number of patient encounters evaluated by the data clearinghouse system for potential reportable events per zip code.

Geographic coverage was examined by using Arc-IMS (ESRI) to plot the zip codes of patients whose data were evaluated by the system, independent of the result (Figure 4). The breadth of geographic coverage provided by the system extended well beyond the Kansas City metropolitan area. The system evaluated patients from all regions of Missouri and Kansas, as well as out-of-state residents.

## Discussion

We evaluated a data clearinghouse approach to biosurveillance using clinical microbiologic laboratory data. Electronic reporting of communicable diseases has been tested in other communities, including Hawaii, Pittsburgh, and Indianapolis (*8*–*10*). Unlike these earlier studies, our data clearinghouse system gathered data from nonaffiliated healthcare providers, applied a centralized data-mapping team to provide efficiency of scale, and delivered data to multiple governmental entities with jurisdictions in a region that crossed state lines.

Following the bioterrorist attack involving anthrax and letters during fall 2001, we developed and deployed this system rapidly. Formal design of the system began in December 2001; the system was fully operational by March 29, 2002. The use of standardized extraction scripts and a centralized data-mapping operation expedited this work. The data clearinghouse system was easy for clinicians to use because data extraction was automatic and did not require them to modify their workflow, unlike other biosurveillance systems that require users to reenter data into a Web page (*12*,*13*). This clearinghouse was also the first reported system to provide laboratory order–trending information to public health organizations.

Analysis of the data clearinghouse system adds to the evidence that electronic reporting of disease can offer substantial benefits to public health. Our finding that electronic reporting improves timeliness is consistent with reports from Hawaii and other areas (*9*). Notably, our system attained a significant improvement in timeliness of detection for *Salmonella* spp., as well as an improvement in underreporting of this pathogen. *Salmonella* and other pathogens tracked by the clearinghouse, including *Shigella*, *E. coli* O157:H7, *Giardia lamblia,* and *Cryptosporidium parvum* are classified by the Centers for Disease Control and Prevention as Class B bioterrorism agents: food or water safety threats. Class B agents have been used in biologic crimes, including the Dulles, Oregon, contamination of salad bars with salmonellae in which 751 people became ill (*14*).

We compared the completeness of the data delivered by electronic reporting to data delivered by conventional means and found improvement for every data element evaluated. Greater completeness of data delivered by electronic reports is a tangible benefit for both healthcare providers and public health workers as it reduces followup requests for additional information. Some fields, address in particular, had low values for completeness even for electronic reporting, which reflects gaps in the information in patient demographic information that is provided by physician offices to clinical laboratories.

The data clearinghouse system reduced underreporting, especially for sexually transmitted diseases and enteric pathogens, conditions that are often underreported (*3*,*5*,*15*). The underreported cases originated from all of the organizations participating in the electronic reporting system (data not shown). Review of a subset of underreported cases suggested three common root causes: misinterpretation of jurisdictional guidelines, misunderstanding of reportable specimen criteria, and use of outdated reporting guidelines by healthcare providers (failure to report pathogens recently added to the guidelines, as occurred with the underreported giardiasis cases).

The data clearinghouse approach to biosurveillance and disease reporting also offers opportunities to relieve healthcare providers from managing multiple jurisdictional relationships, which can lead to underreporting. Through electronic reporting, data were reported from patients residing in Kansas whose laboratory work was performed in Kansas City to both the Department of Health and Environment (with jurisdiction based on the state of residence of the patient) and KCHD (with jurisdiction over the performing laboratory). Reducing underreporting is a critical step in building a threshold-based automated alerting infrastructure because the baseline data gathered by an electronic reporting system is more accurate than that gathered by traditional methods.

During the early stages of this work, we identified a number of confounding issues. For example, a laboratory technician entered a report with the word “No” on one line, followed by “*Bordetella pertussis*” on the next line, with the intent of negating the positive result. This type of data entry error, which led to a few false-positive results propagating into the clearinghouse during the first few weeks of operation, has also been an issue for other electronic reporting systems (*8*). After recognizing this issue, we added context-sensitive logic to clearly flag a reportable isolate preceded by “No” or “Not” as a potential false-positive.

We evaluated whether test orders were predictive of disease incidence. However, in the absence of major outbreaks during the period evaluated, our data were inconclusive. Surveillance of laboratory test orders (i.e., ova and parasite procedure orders) would be useful as an early warning of a water-supply contamination crises, such as that experienced by Milwaukee in 1993 when cryptosporidium contaminated the city water supply and caused >403,000 illnesses (*16*).

Most patients whose results were evaluated by the system resided within approximately 150 km of the Kansas City metropolitan area; however, patients from eastern Missouri, western Kansas, and other U.S. states also were evaluated by the system. The area covered by the clearinghouse crosses the state line that transects the Kansas City metropolitan area. Plans for a national public health infrastructure (e.g., the National Electronic Disease Surveillance System [NEDSS] [*17*]) rely on a process in which patient results are first sent to the state health departments and then forwarded to the Centers for Disease Control and Prevention, currently with identifiable information removed. Reconciling duplicate cases without such information will prove difficult. Systems such as NEDSS that manage data based on politically defined boundaries have inherent inefficiencies that could be rectified by direct reporting to a data clearinghouse. A data clearinghouse can apply jurisdictional filters that control the distribution of reports, while also offering the opportunity to perform rapid analysis of trends across politically defined boundaries.

We considered issues that have proven to be challenging for other electronic reporting projects. In particular, the use of nonstandardized data, subject to errors in either database design or during data entry, created challenges. Overall, we found that the data clearinghouse approach to biosurveillance offers many benefits, including ease and speed of implementation, improved timeliness and completeness of data, efficiency of scale from a central data-mapping operation, and the ability to deliver data to multiple jurisdictions.
